# Long‐term survival of a patient with lung cancer treated with pembrolizumab after recurrent cardiac tamponade

**DOI:** 10.1002/ccr3.6795

**Published:** 2022-12-27

**Authors:** Tsuyoshi Uchida, Hirochika Matsubara, Mamoru Muto, Harunobu Sasanuma, Saya Sugimura, Yuichiro Onuki, Hiroyuki Nakajima

**Affiliations:** ^1^ Department of Thoracic Surgery University of Yamanashi Chuo Japan

**Keywords:** cardiac tamponade, immunotherapy, non‐small cell lung cancer, pembrolizumab, radiotherapy

## Abstract

A 69‐year‐old man with non‐small cell lung cancer presenting with pericardial effusion and rapid progression of dyspnea achieved long‐term disease stabilization after radiation therapy and immunotherapy. This case shows that pembrolizumab may improve prognosis in advanced lung cancer, even when complicated by cardiac tamponade.

## Abstract 2

Patients with advanced lung cancer who develop cardiac tamponade can be treated with pembrolizumab and achieve long‐term prognosis without any serious adverse events.

## 1 INTRODUCTION

Pericardial effusion occurs in patients with advanced non‐small cell lung cancer (NSCLC). However, it is often asymptomatic and detected incidentally during routine examinations.^1^ Thus, routine surveillance is considered essential for diagnosing NSCLC. Immunotherapy with pembrolizumab is considered an effective treatment for advanced lung cancer.^2^ Herein, we report a case of a patient with NSCLC and pericardial effusion that rapidly caused dyspnea; the patient achieved long‐term disease stabilization after radiation therapy and immunotherapy.

## 2 CASE REPORT

### 2.1 History and examination

**FIGURE 1:**
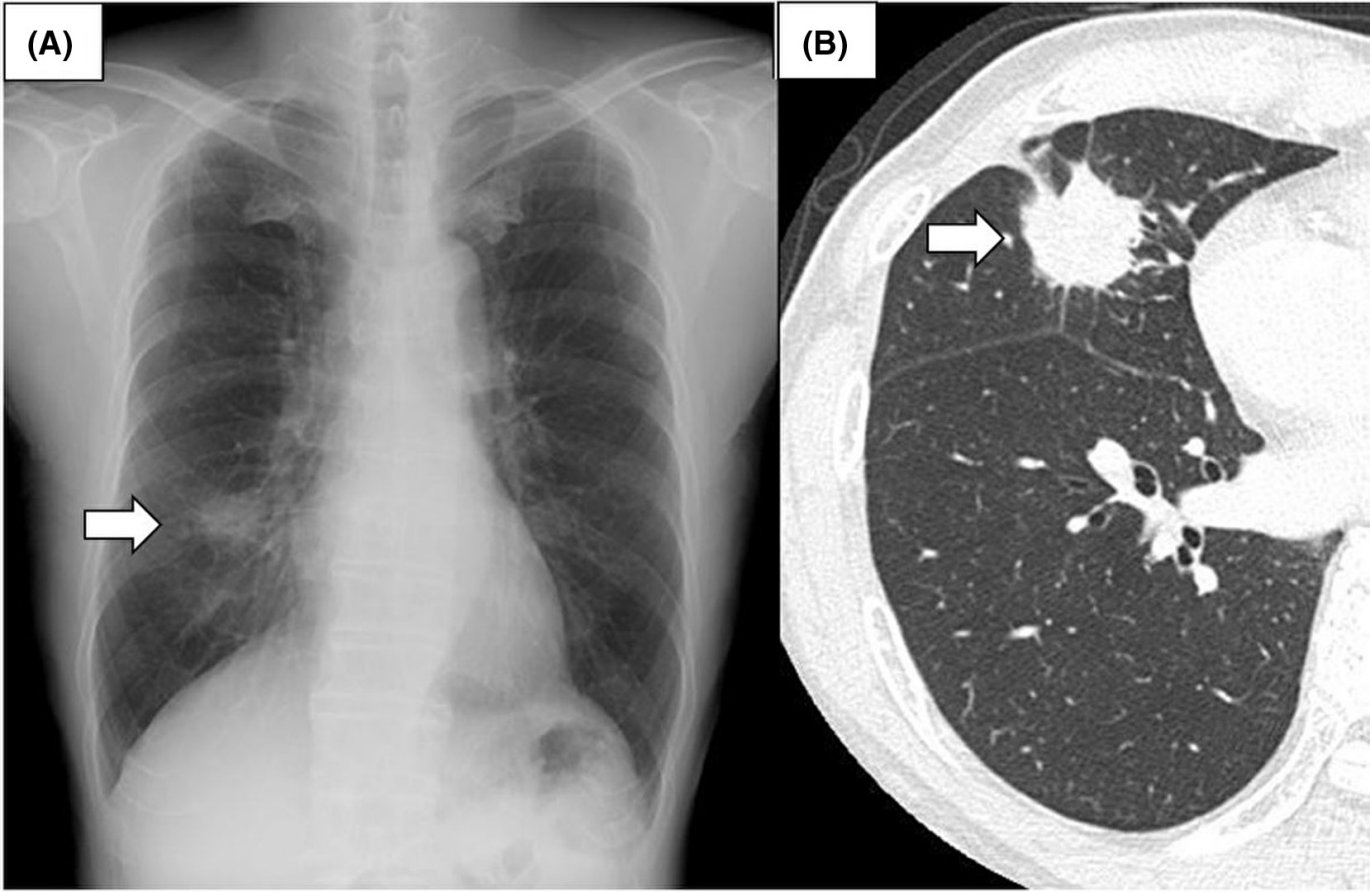
Imaging performed at the time of referral to our hospital showing a pulmonary tumor located in the lower lobe of the right lung. (A) Chest radiography showing a pulmonary nodule in the right lower lung area (white arrow). (B) Transverse view of chest computed tomography showing a pulmonary nodule in the middle lobe of the right lung (white arrow)

**FIGURE 2:**
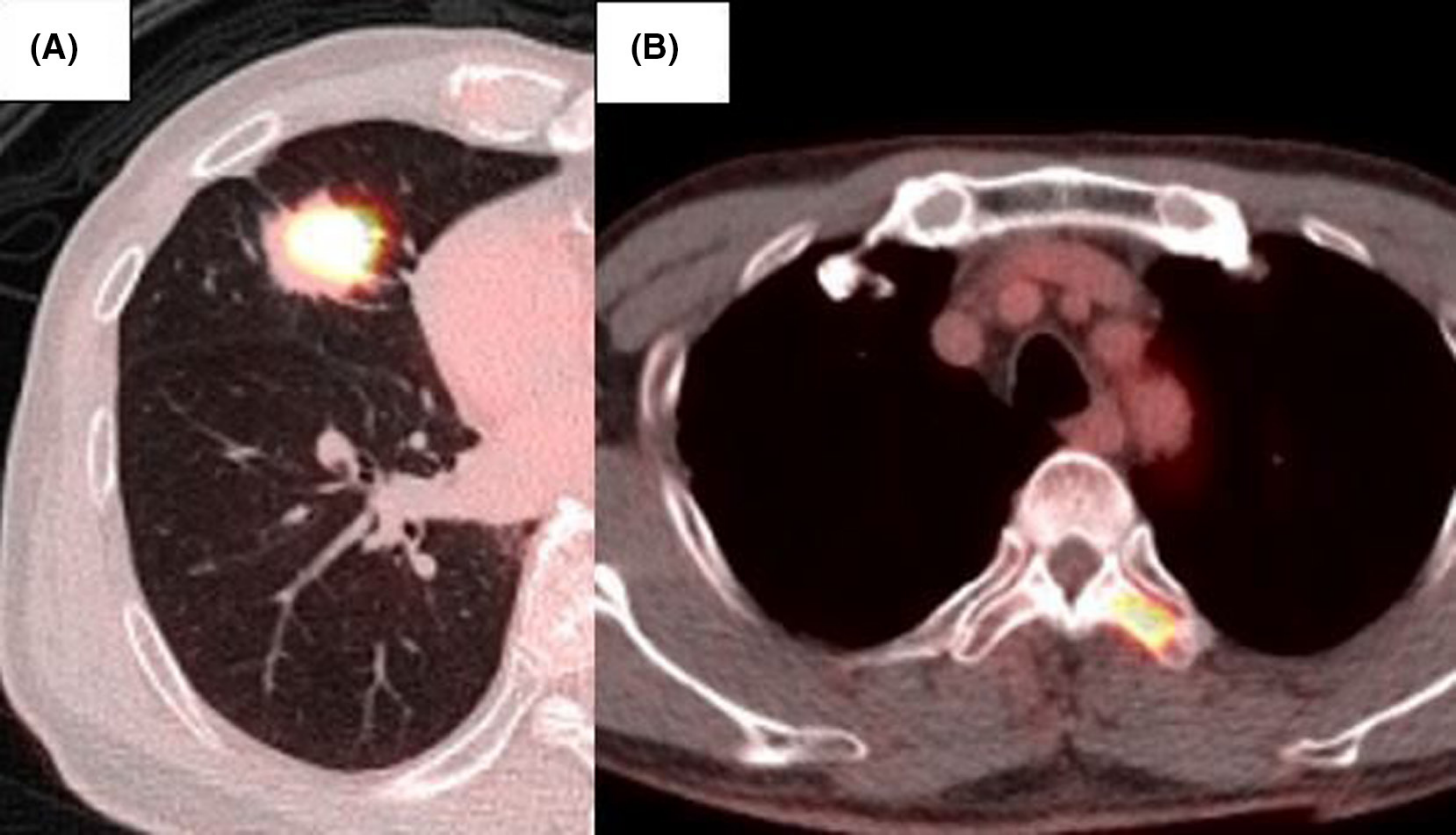
Positron emission tomography surveillance of the mass and discovered bony metastases. (A) Transverse view of the mass with a maximum standardized uptake value (SUVmax) of 7.44. (B) Transverse view at the fourth thoracic vertebra with an SUVmax of 5.32 due to bony metastases of fourth and fifth vertebra

A 69‐year‐old man with a mass noted on chest radiography and computed tomography (CT) was referred to our hospital for surgical resection (Figure 1). Positron emission tomography performed for lung cancer staging revealed that the mass and the fourth thoracic vertebra had maximum standardized uptake values (SUV_max_) of 7.44 and 5.32, respectively (Figure 2). He had previously been catheterized for myocardial infarction; however, no other history was noted. Therefore, a right middle lobectomy was performed to diagnose and resect the pulmonary mass.

Pathological findings indicated that the mass represented pleomorphic lung cancer that had invaded the visceral peritoneum and had metastasized to the 12th lymph node. Programmed death‐ligand 1 (PD‐L1) expression was 90% positive, and the genotype was normal. Thus, the patient was diagnosed with right middle lobe lung cancer (pT2a(pl2)N1M1b stage IVb). The patient received four rounds of chemotherapy with cisplatin and pemetrexed. Six months after the surgery, he underwent salvage radiotherapy to the fourth and fifth thoracic vertebrae for bone metastasis that had not responded to chemotherapy.

**FIGURE 3:**
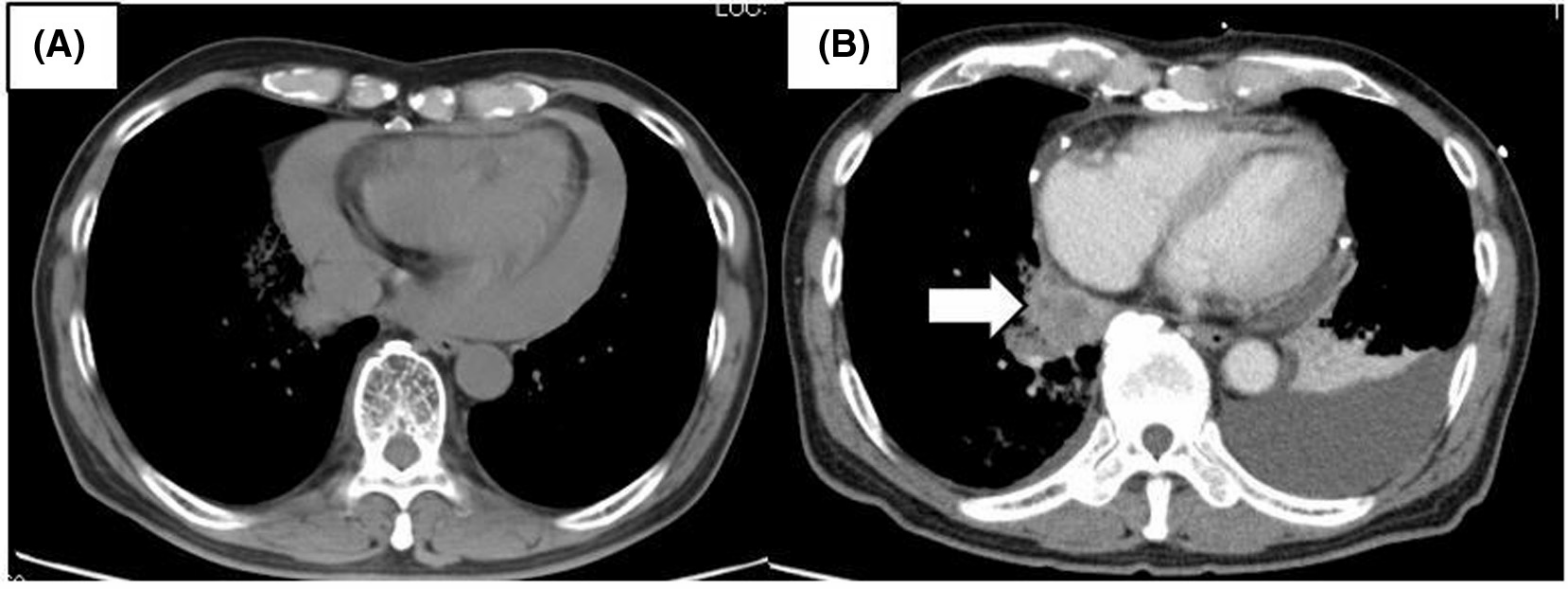
Transverse view computed tomography performed when the patient presented due to progressive dyspnea. (A) Initial chest computed tomography at Fujiyoshida Municipal Hospital showing a pericardial tamponade. Its volume was approximately 150–250 ml. (B) Computed tomography with contrast performed at our hospital showing a tumor (white arrow) and lung cancer recurrence in the right lower lobe involving the inferior pulmonary vein

Two and a half years after the surgery, he experienced dyspnea and fatigue and visited a nearby hospital. At presentation, chest CT revealed pericardial tamponade (Figure 3A).

### 2.2 Differential diagnosis, investigations, and treatment

Bleeding due to pericardial seeding of lung cancer, myocardial perforation, and heart failure were all considered possible causes of cardiac tamponade. Regardless of cause, treatment via drainage of pericardial fluid was necessary. However, drainage alone did not resolve his condition; thus, he was referred to our hospital a week later for surgical treatment. He underwent pericardial drainage under general anesthesia. Intraoperative investigations revealed no obvious source of bleeding, and the drainage tube was removed on postoperative day 2. CT with contrast revealed a recurrent tumor in the right lower lobe involving the inferior pulmonary vein (Figure 3B), which might have caused the hemorrhage or cancerous pericardial effusion. Cytological analysis of the pericardial fluid revealed tumor‐positive cells. Radiotherapy, followed by immunotherapy with pembrolizumab, was used to manage the recurrence.

### 2.3 Outcome and follow‐up

The patient's condition remained stable without evidence of recurrence for 31 months (51 months after the initial surgery) on pembrolizumab therapy.

## 3 DISCUSSION

This case report highlights two significant points. First, cardiac tamponade can occur in patients with lung cancer, even when they are followed up with CT every 3 months. In the present case, the lung cancer had already progressed when surgery was performed; even with CT follow‐up performed every 3 months after adjuvant therapy, cardiac tamponade was not suspected. A small tumor (suspected to be a recurrence of the lung cancer) was discovered retrospectively. No guidelines have been established regarding the duration of follow‐up required for patients with lung cancer. Regarding overall survival, it has been reported that routine surveillance with CT is not superior to symptom‐driven follow‐up^3^; in this case, routine surveillance with CT did not prevent the onset of symptoms.

Second, pembrolizumab may improve prognosis, even in cases of advanced lung cancer with cardiac tamponade. NSCLC continues to be the leading cause of cancer‐related deaths worldwide.^4^ Of the known clinical prognostic factors, tumor stage is the most significant. At stage IIB or higher, the 5 years survival rate is <50%.^2^ In addition, the prognosis of patients with advanced‐stage cancer who develop cardiac effusion is rarely reported to exceed a median of 12 months.^1^ KEYNOTE‐001 revealed a median overall survival of 10.5 months for patients treated with pembrolizumab as the first‐line therapy.^5^ In addition, KEYNOTE‐024 revealed a median overall survival of 26.3 months for patients with a PD‐L1 expression of >50% who were treated with pembrolizumab as the first‐line therapy. Immunotherapy can lead to a variety of immune function‐related adverse events. However, unlike conventional cytotoxic anticancer agents, immunotherapeutic agents are not frequently associated with hematological toxicity. Furthermore, immunotherapy can be easily administered to patients that are in generally poor condition. In our case, the patient's general condition was poor after the onset of cardiac tamponade; however, pembrolizumab enabled prompt treatment initiation. Middleton et al. reported the efficacy of pembrolizumab in patients with lung cancer with a performance status of 2,^6^ but there is no evidence to support that immunotherapy is superior to cytotoxic anticancer agents for lung cancer with poor performance status.

With the exception of having a high PD‐L1 expression, our patient met all the criteria for a poor prognosis. Despite this, with immunotherapy, he has long since passed the reported median survival time (51 months postoperatively), despite developing cardiac tamponade.

## 4 CONCLUSION

Even in cases complicated by cardiac tamponade, pembrolizumab can be a very useful therapy for treating lung cancer. With consideration for the potential of immune system side effects, it can lead to longer prognosis and improvement of quality of life.

## AUTHOR CONTRIBUTIONS

**Tsuyoshi Uchida:** Conceptualization; writing – original draft. **Hirchika Matsubara:** Writing – review and editing. **Mamoru Muto:** Investigation. **Harunobu Sasanuma:** Writing – review and editing. **Aya Sugimura:** Writing – review and editing. **Yuichiro Onuki:** Writing – review and editing. **Hiroyuki Nakajima:** Supervision.

## FUNDING INFORMATION

There are no financial interests or funding to disclose.

## CONFLICT OF INTEREST

The authors declare that they have no conflict of interest.

## CONSENT

Informed consent was obtained from the patient described in this study. A copy of the written consent is available for review by the Editor‐in‐Chief of this journal on request.

## RESEARCH INVOLVING HUMAN PARTICIPANTS

All procedures performed in this case study involving human participants were in accordance with the ethical standards of the institutional and/or national research committee and with the 1964 Helsinki Declaration and its later amendments or comparable ethical standards. The review board of Yamanashi University provided an ethics exemption for this study.

## Data Availability

The data used to support the findings of this case report are available from the corresponding author upon request.
